# *Ficus sycomorus* latex: An efficient alternative Egyptian source for horseradish peroxidase in labeling with antibodies for immunodiagnostic kits

**DOI:** 10.14202/vetworld.2018.1364-1370

**Published:** 2018-10-01

**Authors:** Azza M. Abdel-Aty, Mohamed Belal Hamed, Abdul Aziz M. Gad, Amr E. El-Hakim, Saleh A. Mohamed

**Affiliations:** Molecular Biology Department, National Research Centre, Dokki, Cairo, Egypt

**Keywords:** conjugation, *Ficus sycomorus*, horseradish peroxidase, immunodiagnostic assays, latex

## Abstract

**Aim:**

In view of various peroxidase applications, the searching for new sources of unique peroxidase properties is highly desirable. The present study aims to evaluate the efficiency of the peroxidase of locally grown sycamore latex (POL) for conjugation with antibodies and to study the conjugate optimal conditions, storage stability, and affinity toward different substrates as compared with commercial horseradish peroxidase (HRP).

**Materials and Methods:**

Anti-mouse antibodies were prepared in rabbits and purified by protein A sepharose affinity column chromatography. The POL and HRP conjugates were prepared by one-step glutaraldehyde coupling method. The reactivity of the prepared conjugates was examined using the enzyme-linked immunosorbent assay (ELISA). The optimal enzymatic conditions, storage stability, and affinity toward substrates were also determined for both the conjugates.

**Results:**

The POL showed higher percent recovery (98%) than HRP (78%) over the initial activity after conjugation process. The POL and HRP conjugates showed ELISA titers of 1:120 and 1:80, respectively, demonstrating high binding affinity of POL-conjugate. The POL-conjugate showed high thermal stability up to 70°C compared with HRP-conjugate up to 40°C. After conjugation, POL had wide pH stability (5.0-8.0) compared with HPR (4.5-6.0). Both of the prepared conjugates had a high affinity toward the substrates used in immunoassays with lower K_m_ values. The POL-conjugate showed high storage stability for its enzymatic activity and ELISA titer compared with HRP-conjugate after 1 year at −20°C.

**Conclusion:**

The POL of *Ficus sycomorus* latex is an efficient source for labeling antibodies and could be utilized in immunodiagnostic kits.

## Introduction

Peroxidases (EC.1.11.1.7) are hydrogen peroxide decomposing enzymes associated with oxidation of the broad range of phenolic and non-phenolic substrates. Plant peroxidases have an essential physiological role in the growth and development of plant throughout its life cycle. Due to the versatility of peroxidases during reaction and their ubiquitous nature, they have potential applications in various immunological, medicinal, biotechnological, and industrial sectors [1-3].

Latex is a natural plant polymer flowed from various plant parts after having a tissue injury. It is a complex mixture of phytochemicals and unique enzymes that protect the plant against bacteria, fungi, viruses, and feeding insects [4]. Latex peroxidases have unique properties to perform a protective role against possible oxidative damage which begins after plant is wounded [5].

*Ficus sycomorus* (Family: Moraceae) are short trees native to Africa. Its fruits (figs) widely used as a food and medicine all over the world [6-8]. Fig latex has potential therapeutic effects against several diseases [9]. Further, we previously purified and characterized peroxidase enzyme from locally grown sycamore fig latex (POL). This enzyme showed potent properties such as high thermal stability, broad pH optimum, and high affinity toward substrates commonly used in immunodiagnostic kits [10]. Moreover, POL was effectively decolorized industrially important synthetic and natural dyes [11].

From this standpoint, the present study aims to evaluate the efficiency of the POL enzyme, from locally grown sycamore fig latex, for conjugation with antibodies, and to study the conjugate optimal enzymatic conditions, its storage stability, its affinity toward different substrates, and its utility in enzyme-linked immunosorbent assay (ELISA) as compared with commercial horseradish peroxidase (HRP).

## Materials and Methods

### Ethical approval

All the experimental protocols described in this study were performed in accordance with the recommendations of the Ethical Committee of National Research Centre (NRC), Egypt.

### Materials

HRP, 2,2’-azino-bis (3-ethylbenzo-thiazoline-6-sulfonic acid) (ABTS), 4-chloro-1-naphthol (4C-1N), 3,3`,5,5`tetramethylbenzidine (TMB), o-phenylenediamine dihydrochloride (OPD), guaiacol, hydrogen peroxide, protein A Sepharose CL-4B column, and monoclonal mouse anti-albumin IgG were purchased from Sigma Scientific Services Co. All other chemicals and reagents were of analytical grade. The buffers were prepared according to Gomorie [12], and the final pH was checked by pH meter. The POL peroxidase was previously purified from locally grown *F. sycomorus* fruit latex [10].

### Peroxidase assay

Peroxidase activity was carried out according to Miranda *et al*. [13]. The reaction mixture contains in 1 ml: 8 mM H_2_O_2_, 40 mM guaiacol, 20 mM sodium acetate buffer, pH 5.5, and 0.1 ml enzyme sample. The change in absorbance was measured at 470 nm and room temperature (28-30°C) using a spectrophotometer. One unit of peroxidase activity was defined as the amount of enzyme which increases the optical density (OD) 1.0 per min under standard assay conditions.

### Preparation and purification of rabbit anti-mouse IgGs (AM IgGs)

AM IgGs were prepared in rabbits according to Hudson and Hay [14]. Two rabbits (each 1.5±0.11 kg) were immunized by four subcutaneous injections of 60 µg of mouse IgG dissolved in 0.5 ml saline at 3-week intervals. The first dose was mixed with 0.5 ml of complete Freund’s adjuvant. The subsequent doses were mixed with 0.5 ml of incomplete Freund’s adjuvant. The rabbits were bled, and the antisera were separated, pooled, and purified using the protein A Sepharose CL-4B column (1.6 cm×4 cm) equilibrated and washed with 0.1 M Tris-HCl pH, 7.4. The adsorbed proteins were eluted with 0.1M glycine-HCl, pH 2.9, at a flow rate of 60 ml/h and monitored at 280 nm. The active fractions that contained the purified AM IgGs were concentrated by 75% ammonium sulfate precipitation.

### Coupling of the AM IgGs with POL and HRP

One-step coupling method was used according to Tresca *et al*. [15] with slight modifications in coupling ratio and dialysis buffer. The peroxidase enzyme (1.5 mg) and AM IgGs (0.75 mg) in 2 ml of 0.1 M phosphate buffer, pH 6.8, were incubated for 2 h at room temperature (28-30°C) with 50 µl of 1% solution of glutaraldehyde under gentle stirring, and then, the mixture was dialyzed several times against 20 mM Tris-HCl buffer, pH 7.4, included 5 mM CaCl_2_ and 150 mM NaCl. The solution was centrifuged at 16,000× *g* for 20 min at 4°C, and the precipitate was removed.

### ELISA

The reactivity of the prepared conjugates was examined in 96-well microtiter plates using the ELISA test described by Ricoux *et al*. [16]. Briefly, each well was coated overnight with 100 µl of monoclonal mouse anti-albumin IgG (0.5 µg protein/well) dissolved in coating buffer (50 mM sodium carbonate buffer, pH 9.6). Non-specific sites were blocked for 1 h by the addition of 100 µl of the blocking solution (2% gelatin dissolved in coating buffer) at 37°C. Each well was incubated with 100 µl of serum albumin (1 µg/well) dissolved in washing buffer (0.01 M PBS, pH 7.4 containing 0.05% Tween-20) at 37°C for 1 h. Serial dilutions (100 µl) of the prepared conjugate dissolved in washing buffer were added and incubated at 37°C for 1h. Finally, 100 µl of substrate buffer (0.33 mg OPD/ml dissolved in citrate buffer, pH 4.5, containing 0.04% H_2_O_2_) was added to each well, then the reaction was stopped after 20 min by the addition of 20 µl of a 1:20 dilution of sulfuric acid and the absorbance was determined at 490 nm with ELISA reader. All the values were recorded in duplicate. A standard curve between log conjugate dilution and log OD was plotted. The dilution that gives 0.5 OD. at 490 nm was taken as the ELISA titer.

### Characterization of conjugated enzyme properties

The peroxidase activity standard assay was used to monitor the characterization of both the conjugated enzymes. The optimum pH was examined using 20 mM of sodium acetate, sodium phosphate, and Tris-HCl buffers over a pH range of 3.5-6.0, 6.0-7.0, and 7.0-9.0, respectively. To examine the pH stability, the enzyme was incubated at the same pH buffers and room temperature (28-30°C) for 2 h before substrate addition and measurement of the residual activity. The optimum temperature was examined by incubating the reaction mixture at different temperatures ranging from 10°C to 90°C. The effect of temperature on the enzyme stability was examined by preincubating the enzyme for 30 min in different temperatures ranging from 10°C to 90°C before substrate addition, followed by cooling in ice bath, and the remaining activity was determined.

### Protein determination

Protein concentration was quantified by the method of Bradford [17] using bovine serum albumin as a standard.

### Statistical analysis

The results are reported as mean±standard error for at least 4 times’ experiments.

## Results and Discussion

Among peroxidases, HRP is commonly used for many analytical and industrial purposes. However, other plant peroxidases possessed better substrate specificities, broad pH and thermal stability, yield, and economic feasibility and could be optional for HRP [1]. The peroxidase of sweet potato has high specific activity and unique electrochemical characters as well as easily available; therefore, it is used as a biosensor [18]. Tobacco peroxidase was immobilized to graphite electrodes and used for the detection of aromatic compounds [19]. The peroxidase of spring cabbage has a strong preference toward various substrates, high thermal and pH stability, and low costs of extraction, so it is used as a bioelectrocatalysis [20]. Turnip root peroxidases have been used for uric acid detection kits [21]. The searching for new sources of unique peroxidase properties such as high stability toward temperature, pH, and heavy metals, and high affinity toward broad substrates is highly desirable. These potent properties have been previously detected in the purified peroxidase (POL) of *F. sycomorus* fruit latex [10]. Further, the peroxidase has great ability to produce stable chromogenic products, so it is a suitable enzyme in various diagnostic kits based on enzyme-conjugated antibody technology [1,22]. Therefore, the present study is directed to prepare POL-conjugate with high storage stability and binding affinity toward substrates compared with commercial HRP-conjugate.

### Purification of rabbit IgGs

**Figure-1:**
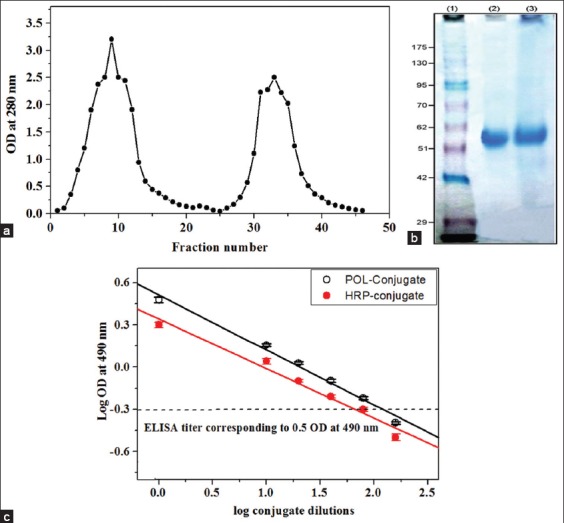
(a) Affinity chromatography of rabbit anti-mouse IgGs (AM IgGs) on a protein A Sepharose column (1.6 cm×4 cm). The unbound proteins were washed out by 0.1M Tris-HCl, pH 7.4, while the bound proteins were eluted with 0.1M glycine-HCl, pH 2.9 at a flow rate of 60 ml/h. (b) Sodium dodecyl sulfate-polyacrylamide gel electrophoresis (SDS_PAGE) of the purified AM IgGs. Lane (1): MW marker, Lanes (2 and 3): 5 and 10 µg of the purified AM IgGs, respectively. (c) Enzyme-linked immunosorbent assay (ELISA) titration curve of the prepared sycamore latex peroxidase (POL) and horseradish peroxidase (HRP) conjugates. Log_10_ conjugate dilution versus Log_10_ *A*_490_ is presented. The values represent mean±standard error (n=4).

The anti-immunoglobulins needed for conjugation with either POL or HRP peroxidases were prepared by injection of mouse IgG in rabbits using a traditional protocol according to Hudson and Hay [14]. Several methods were reported for the purification of serum IgG; however, the affinity chromatography method is considered as a simple, one-step, and highly efficient method for IgG purification [23]. Therefore, the prepared sera were collected, pooled, and applied on a protein A Sepharose affinity column. In Figure-1a, the chromatographic profile of the protein A Sepharose column showed two protein peaks, the second peak (28-40 fractions) was pooled, concentrated by 75% ammonium sulfate precipitation and designated as a rabbit AM IgGs. A quantity of 24 mg of AM IgGs was yielded from 4 ml of rabbit antiserum. The purity of AM IgGs was confirmed by sodium dodecyl sulfate-polyacrylamide gel electrophoresis (Figure-1b). Similarly, a large amount of highly purified yield of IgG was previously reported when the protein A sepharose affinity method was used [24].

### Conjugation of purified POL and HRP to AM IgGs

Enzyme-antibody conjugate is most often prepared by cross-linking between the enzyme and the antibody either by their functional groups or sugar moieties attached to one of the proteins. Therefore, the coupling with homobifunctional reagent such as glutaraldehyde is common [25,26]. Table-1 shows the characteristics of POL and HRP peroxidases before and after conjugation process. At the beginning, either of POL or HRP peroxidase (1.5 mg protein/ml) with total activity (5000 and 8070 U) and specific activity (3333 and 5380 U/mg protein), respectively, were conjugated to 0.75 mg of AM IgGs in a ratio of 2:1 (w/w). After conjugation, the POL-IgG conjugate (2 mg protein/ml) had total activity and specific activity of 4890 U and 2445 U/mg protein compared with the HRP-IgG conjugate (2.2 mg protein/ml) 6250 U and 2802 U/mg protein, respectively. Further, the POL-conjugate showed higher percentage recovery (98%) than the HRP-conjugate (78%) over the initial activity. These results indicated that most of the POL enzymatic activities were retained after the coupling process, demonstrating that this method did not alter the functionality of the POL enzyme. However, the percentage recovery of Turnip-peroxidase (TPOD) coupled with antibodies by glutaraldehyde method was 58.5% [27]. The peroxidase of *Brassica oleracea gongylodes* also retained 54% of its activity after conjugation [28]. Here, to evaluate the binding affinity of the peroxidase enzyme to antibody and to quantify the concentration of antibody in each prepared conjugate, ELISA experiment was designed. The POL and HRP conjugates showed ELISA titer of 1:120 and 1:80, respectively (Figure-1c and Table-1), suggesting that the POL-conjugate has greater binding affinity than the HRP-conjugate. This is higher than a TPOD-conjugate of turnip which has a reported ELISA titer of 1:10 [27]. Differences in ELISA titers among conjugates were attributed to the differences of coupling methods, coupling ratio between antibody and the enzyme, the concentration of coupling agent and optimization conditions during conjugation process [28,29].

**Table-1:** Conjugation of the purified POL and HRP to AM IgG.

Sample	Protein (mg)	Activity (U)	Specific activity (U/mg)	Recovery (%)	ELISA titer
POL	1.5	5000	3333	100	-
POL-IgG	2	4890	2445	98	1:120
HRP	1.5	8070	5380	100	-
HRP-IgG	2.2	6250	2802	78	1:80

ELISA=Enzyme-linked immunosorbent assay, HRP=Horseradish peroxidase, AM IgG=Anti-mouse IgG, POL= Sycamore latex peroxidase

### Characterization of the POL and HRP conjugates

**Figure-2:**
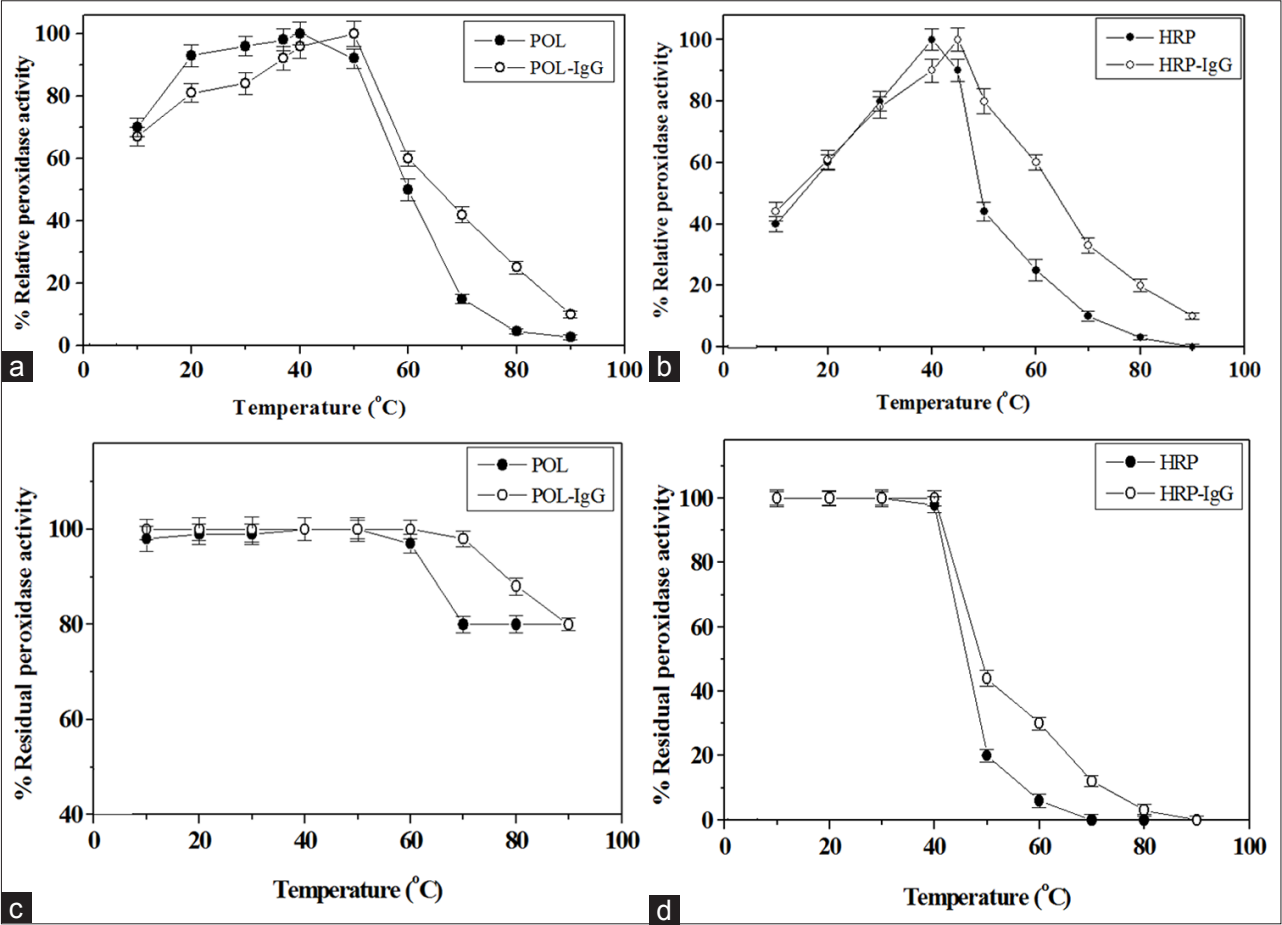
Typical profiles for the assessment of the optimum temperature (a and b) and thermal stability (c and d) of the sycamore latex peroxidase (POL) and horseradish peroxidase (HRP) before and after conjugation process. The values represent mean±standard error (n=4).

Both the POL and HRP had temperature optima at 40°C, and they increased to 50 and 45°C after conjugation, respectively (Figure-2a and b). Further, the purified POL and POL-conjugate showed high thermal stability since no loss of activity was recorded up to 60 and 70°C, respectively. However, the HRP and HRP-conjugate showed similar thermal stability up to 40°C (Figure-2c and d). The peroxidase resistance to temperature depends on the source of the enzyme and assay conditions [10]. Turnip TPOD-conjugate showed thermal stability up to 40°C, and the activity decreased with increasing temperature up to 65°C [27]. The peroxidase-conjugate of *B. oleracea gongylodes* possessed thermal stability up to 50°C [28].

**Figure-3:**
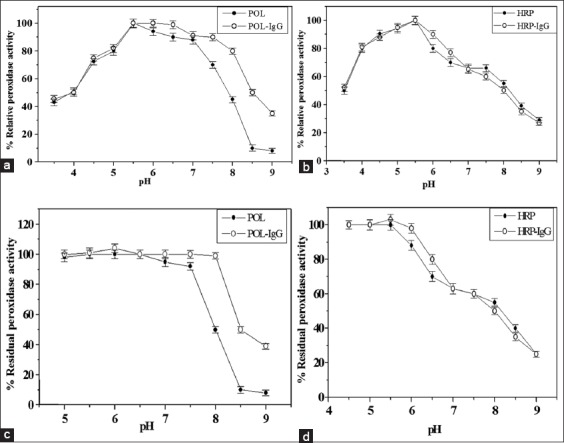
Typical profiles for the assessment of the optimum pH (a and b) and pH stability (c and d) of the sycamore latex peroxidase (POL) and horseradish peroxidase (HRP) before and after conjugation process. The values represent mean±standard error (n=4).

The POL, HRP, and HRP-conjugate possessed the same pH optima at 5.5, while it became a broad (5.5-6.5) in case of POL-conjugate (Figure-3a and b). Both of the purified TPOD and TPOD-conjugate prepared by glutaraldehyde method exhibited the same pH optima at 3.5 [27]. The pH stability of the POL and HRP was broad (5-7 and 4.5-5.5), while it was increased to 5-8 and 4.5-6, respectively, after conjugation (Figure-3c and d). The pH stability of the peroxidase of *B. oleracea gongylodes* improved after conjugation [28]. It was observed that the POL relative peroxidase activity was higher than the HRP activity in most characterization assays, while the HRP possessed slightly higher relative peroxidase activity than POL at pH ranges (3.5-5.0 and 8.0-9.0). Table-2 summarizes the most measured parameters of the prepared conjugates compared to POL and HRP enzymes.

**Table-2:** Physical and kinetic studies of the POL and HRP before and after conjugation with AM-IgG.

Sample	Optimum pH	pH stability	Optimum temperature °C	Thermal stability °C	*K_m_* (mM substrates)			

					ABTS	4C-1N	OPD	TMB
POL	5.5	5.0-7.0	40	10-60	4.5±0.5	5.0±0.6	5.8±0.4	7.0±0.8
POL-IgG	5.5-6.5	5.0-8.0	50	10-70	3.8±0.63	4.3±0.52	4.8±0.5	6.4±0.4
HRP	5.5	4.5-5.5	40	10-40	4.3±0.67	6.2±0.4	5.0±0.32	4.0±0.7
HRP-IgG	5.5	4.5-6.0	45	10-40	4.0±0.52	5.9±0.67	4.6±0.41	3.7±0.4

Values are presented as means±standard error (n=4). HRP=Horseradish peroxidase, POL= Sycamore latex peroxidase, ABTS=2,2’-Azino-bis (3-ethylbenzo-thiazoline-6-sulfonic acid), OPD=O-phenylenediamine dihydrochloride, 4C-1N = 4-chloro-1-naphthol, TMB = 3,3`,5,5`tetramethylbenzidine, AM-IgG=Anti-mouse IgG

The effect of different concentrations of substrates (ABTS, 4C-1N, OPD, and TMB) that commonly used in immunodiagnostic kits on the prepared conjugates was also studied. In Table-2, both the POL and HRP conjugates exhibited lower K_m_ values using ABTS, 4C-1N, OPD, and TMB (3.8, 4.3, 4.8, and 6.4 and 4.0, 5.9, 4.6, and 3.7 mM) than that of POL and HRP (4.5, 5.0, 5.8, and 7.0 and 4.3, 6.2, 5.0, and 4.0 mM), respectively. It is likely that the prepared conjugates retained their high affinity toward the substrates which used in immunoassays. Lower K_m_ value using ABTS was previously reported for the TPOD-conjugate [27]. The peroxidase-conjugate of *B. oleracea gongylodes* also showed low K_m_ values to phenol and BCP and BTB dyes [28].

**Figure-4:**
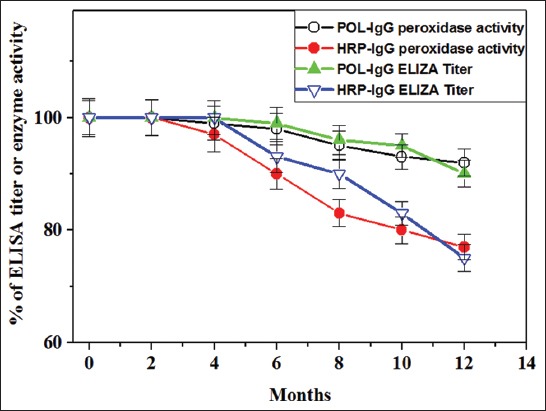
Storage stability assessment of the prepared sycamore latex peroxidase (POL) and horseradish peroxidase (HRP) conjugates during 1 year at −20°C. The values represent mean±standard error (n=4).

The storage stability of the prepared conjugates was evaluated by measuring both of the ELISA titer and enzyme activity percentage during 12 months at −20°C. In Figure-4, the POL-conjugate showed 100% stability for its enzyme activity and ELISA titer up to 6 months compared with HRP-conjugate 90%. Furthermore, only 8-10% activity and binding titer of the POL-conjugate were lost up to 12 months compared with HRP-conjugate (23-25%), respectively. Moreover, a strong correlation between the enzyme activity and binding titer of both conjugates was observed. The HRP-conjugate using cyanuric chloride showed no change in ELISA titer during 12 months when it stored at −70°C [29]. The TPOD-conjugate of turnip showed 20% loss of enzyme activity over 6 months at −20°C [27]. The HRP labeled by anti-camel IgG showed stable activity for 1 year at −20°C [30]. The peroxidase-conjugate of *B. oleracea gongylodes* retained 95% of enzyme activity after 2 months when stored at −20°C [28].

## Conclusion

From the current study, *F. sycomorus* latex peroxidase (POL) and HRP conjugates were prepared using glutaraldehyde coupling method. In comparison with the prepared HRP-conjugate, the POL-conjugate retained most of its enzymatic activity and showed greater binding affinity. Further, the POL enzyme parameters (temperature and pH optima and thermal and pH stability) were improved after conjugation. The POL-conjugate showed high storage stability for 1 year at −20°C. Both of the prepared conjugates retained their high affinity toward the immunodiagnostic kit substrates with low K_m_ values. Consequently, the POL peroxidase enzyme is an efficient Egyptian source in labeling with antibodies for immunodiagnostic kits.

## Authors’ Contributions

AMA, MBH, AEE, and SAM had the original idea for the study and carried out the design. AMA, MBH, and AAMG were responsible for data analysis, data cleaning, and writing the manuscript. The final draft manuscript was revised by all authors.All authors read and approved the final manuscript.
